# Single-cell RNA sequencing of peripheral blood reveals immune cell dysfunction in premature ovarian insufficiency

**DOI:** 10.3389/fendo.2023.1129657

**Published:** 2023-05-08

**Authors:** Caihong Zhang, Dong Yu, Yue Mei, Shanrong Liu, Huijing Shao, Qianqian Sun, Qiong Lu, Jingjing Hu, Hang Gu

**Affiliations:** ^1^ Department of Obstetrics and Gynecology, The First Affiliated Hospital of Naval Medical University, Shanghai, China; ^2^ Department of Precision Medicine, Translational Medicine Research Center, Naval Medical University, Shanghai, China; ^3^ Shanghai Key Laboratory of Cell Engineering, Shanghai, China; ^4^ Department of Laboratory Diagnostics, The First Affiliated Hospital of Naval Medical University, Shanghai, China; ^5^ Department of Obstetrics and Gynecology, Shanghai First Maternity and Infant Hospital, Tongji University School of Medicine, Shanghai, China

**Keywords:** premature ovarian insufficiency, single-cell RNA sequencing, PBMC, immune cell, biological analysis

## Abstract

**Background:**

Premature ovarian insufficiency (POI) is one of the most common causes of female infertility and the etiology is highly heterogeneous. Most cases are idiopathic and the pathogenesis remains unclear. Previous studies proved that the immune system plays a crucial role in POI. However, the precise role of immune system remains unclear. This study aimed to analyze the characteristics of peripheral blood mononuclear cells (PBMC) from patients with POI by single-cell RNA sequencing (scRNA-seq) and to explore the potential involvement of immune response in idiopathic POI.

**Methods:**

PBMC was collected from three normal subjects and three patients with POI. PBMC was subjected to scRNA-seq to identify cell clusters and differently expressed genes (DEGs). Enrichment analysis and cell-cell communication analysis were performed to explore the most active biological function in the immune cells of patients with POI.

**Results:**

In total, 22 cell clusters and 10 cell types were identified in the two groups. Compared with normal subjects, the percentage of classical monocytes and NK cells was decreased, the abundance of plasma B cells was increased, and CD4/CD8 ratio was significantly higher in POI. Furthermore, upregulation of *IGKC, IFITM1, CD69, JUND* and downregulation of *LYZ, GNLY, VCAN*, and *S100A9* were identified, which were enriched in NK cell-mediated cytotoxicity, antigen processing and presentation, and IL-17 signaling pathway. Among them, *IGHM* and *LYZ* were respectively the most significantly upregulated and downregulated genes among all cell clusters of POI. The strength of cell-cell communication differed between the healthy subjects and patients with POI, and multiple signaling pathways were assessed. The TNF pathway was found to be unique in POI with classical monocytes being the major target and source of TNF signaling.

**Conclusions:**

Dysfunction of cellular immunity is related to idiopathic POI. Monocytes, NK cells, and B cells, and their enriched differential genes may play a role in the development of idiopathic POI. These findings provide novel mechanistic insight for understanding the pathogenesis of POI.

## Introduction

Premature ovarian insufficiency (POI), also termed premature ovarian failure (POF), is characterized by the cessation of ovarian function before 40 years of age (1). POI is one of the most common causes of female infertility and increases the risk of cardiovascular diseases and osteoporosis (2). European Society of Human Reproduction and Embryology (ESHRE) diagnostic criteria define POI as oligo/amenorrhea for at least 4 months and elevated gonadotropins (FSH > 25 mIU/mL) on two occasions over 4 weeks (3). Approximately, 1% to 5% of reproductive-aged women suffer from POI (4). Studies have demonstrated that the etiology of POI is highly heterogeneous and various factors, including chromosomal abnormality, gene mutation, autoimmunity, environmental factors, and iatrogenic factors, are involved (5) Among them, chromosomal abnormalities explain 10% to 15% of POI cases and genetic disorders account for 20% to 25% of cases (6). However, the majority of cases remain unexplained, known as idiopathic POI (7).

Studies have shown that 10% to 55% of patients with POI are complicated with autoimmune diseases, including type 1 diabetes, autoimmune thyroiditis, systemic lupus erythematosus (SLE), psoriasis, rheumatoid and arthritis (RA) (8). The human ovary can be the target of autoimmunity, which can cause ovarian dysfunction (9). The presence of oophoritis and circulating autoantibodies has been reported in a subset of women with autoimmune POI (10).

It has been proven that cellular immune dysfunction is related to ovarian physiology, and large numbers of immune cells invade ovarian tissue in the mice model of CTX-induced POI (11). The proportion of T lymphocytes in thymectomy-induced POI mice was unbalanced (12). It was shown that patients with POI have a reduced number of CD4+/CD25+ Tregs in their peripheral blood (13–15). Currently, more and more studies are focusing on the protective effect of stem cells on POI (16–18). Human umbilical cord blood mesenchymal stem cells (hUCMSCs) can restore ovarian function in mice with chemically-induced POI. hUCMSCs regulate Th1/Th2 imbalance and control NK cell-mediated response (16). MSCs can obtain immunoregulatory properties by regulating the differentiation of macrophages and T cells and controlling their cytokines in POI (17). Another study revealed that human placenta mesenchymal stem cells (hPMSCs) restored ovarian function in mice with autoimmune POI by modulating Treg cell function (18). Immune cell dysfunction can contribute to ovarian dysfunction. However, the mechanism of idiopathic POI is not well understood. Due to potential risks, ovarian biopsy is not routinely recommended. Meanwhile, there is a lack of reliable and specific predictors for idiopathic POI in clinics.

Over the past decades, based on NGS, numerous genes have become candidates for POI, whereas only a few genes have been proven to cause POI. These genes were related to primordial germ cell migration and proliferation (NANOS3), cell death (PGRMC1 and FMR1), oocyte-specific transcription factors (FIGLA and NOBOX), other transcription factors affecting follicular genesis (NR5A1, WT1, and FOXL2), transforming growth factor-β superfamily (BMP15 and GDF9), and hormone and receptors (FSHR, AMH, and AMHR2). Until now, no single factor could explain POI (19) Traditional bulk transcriptome sequencing detects differences in gene expression among samples, whereas single-cell transcriptome sequencing, a new technology for high-throughput sequencing analysis, can reflect the heterogeneity of cells and simultaneously reveal gene expression characteristics in different cells from a limited amount of sample (20). Thus, cell types can be clustered by transcriptome analysis rather than membrane surface markers (21). In addition, numerous cell types can be characterized between control and case samples, which contributes to understanding the etiology of POI.

Peripheral blood lymphocyte subsets are important parameters for monitoring the immune response. Lymphocytes and their subsets, including NK cells, B cells, and T cells, are mainly responsible for regulating host immunity (22). To date, most studies on lymphocyte subsets in patients with POI have focused on the abundance of each subset of lymphocytes (23). However, their role, function, and biological significance are poorly understood. Hence, this study was performed to investigate the cell composition and function in the PBMCs of POI patients and healthy individuals. The results showed that the composition of several cell subsets, mainly monocytes, NK cells, and B cells, was significantly different in patients with POI. DEGs were identified in the cell subsets, which were mainly involved in the regulation of inflammation and immune response. TNF signaling pathway was identified as the unique pathway in the monocytes of patients with POI. This study illuminates the involvement of PBMC and a set of candidate markers in the pathogenesis of POI.

## Materials and methods

### Participants

All participants, aged between 30 and 40 years, were recruited from the Endocrinology Clinic of Changhai Hospital from June 2021 to October 2021. Patients with POI (n=3) and healthy control (HC, n=3) women with normal ovarian reserve were recruited. According to the ESHRE and Chinese guidelines, the eligibility criteria for POI included secondary amenorrhea for at least 4 months and serum basal FSH > 25 IU/L (on two occasions with more than 1 month interval) before 40 years of age. Women with chromosomal abnormalities, known gene mutations, history of ovarian surgery, radiotherapy or chemotherapy, recurrent spontaneous abortion, endometriosis, autoimmune diseases, and infection within three months before enrollment were excluded. This study was approved by the Institutional Review Board of Changhai Hospital. All participants signed the written informed consent forms.

### Single-cell RNA-seq processing

The preparation and cell suspension of PBMC were performed as previously described (24). Cell capture, cDNA amplification, and library preparation were performed using Chromium Single Cell 3’ Library and Gel Bead Kit v2 (10x Genomics). Then, the libraries were sequenced on the Illumina Nova Seq 6000 platform with a 150-bp paired-end mode.

### Cell quality control and cell clustering

The Cell Ranger software pipeline (version 5.0.0) provided by 10×Genomics was used to preprocess the raw sequencing data across samples, producing a matrix of gene counts versus cells. The gene-barcode matrix of unique molecular identifier (UMI) counts was then analyzed with Seurat (version 4.0.0) (25) for quality control, normalization, dimensional reduction, batch effect removal, clustering, and visualization. To remove low-quality cells and multiple captures, the following criteria were applied: 1- Cells with UMI/gene numbers more than the limit of median value +/- 2 fold of median absolute deviations were filtered out, assuming a Guassian distribution of each cells’ UMI/gene numbers. 2- We discarded low-quality cells where >10% of the counts belonged to mitochondrial genes. 3- We removed the potential duplicates identified by the Doublet Finder package (version 2.3.0) (25). The count matrix was log-normalized, and the top 3000 most variable genes were identified for dimensional reduction. Most of the variable genes were selected using the “Find variable Genes” function. Principal component analysis (PCA) was performed to reduce the dimensionality with “Run PCA” function in Seurat. Graph-based clustering was performed to cluster cells according to their gene expression profile using the “Find Clusters” function. The “Find All Markers” function was used to identify marker genes of each cluster. The R package Single R (26), a novel computational method for unbiased cell type recognition of scRNA-seq was used to independently infer the cell of origin for each single cell and identify cell types based on the reference transcriptomic dataset ‘Human Primary Cell Atlas’ (27).

### Differential expression and functional enrichment analysis

A pseudo-bulk expression profile was generated by summing the UMI counts of all cells with the same combination of cell type and sample. We then performed differential expression analysis for each cell type on the sample level using the R package DESeq2. The R package cluster Profiler (v.3.16.0) was used for function over-representation analyses of DEGs with FDR < 0.05.

DEGs were identified using the “Find Markers” function in Seurat. P value < 0.05 and |log_2_ fold change| > 2 was set as the threshold for significantly differential expression. GO enrichment and KEGG pathway enrichment analyses of DEGs were performed using the R package cluster Profiler (28) and based on the hypergeometric distribution.

### Cell-cell communication analysis

Cell Chat was utilized to infer and analyze global cell-cell communications from scRNA-seq data. For cells in HC or POF, gene expression data for corresponding cells were utilized as input, and cell-cell communication probability was calculated between interactions of ligands.

## Results

### Analysis of the heterogeneity of PBMC populations by single cell RNA-Seq

**Figure 1:**
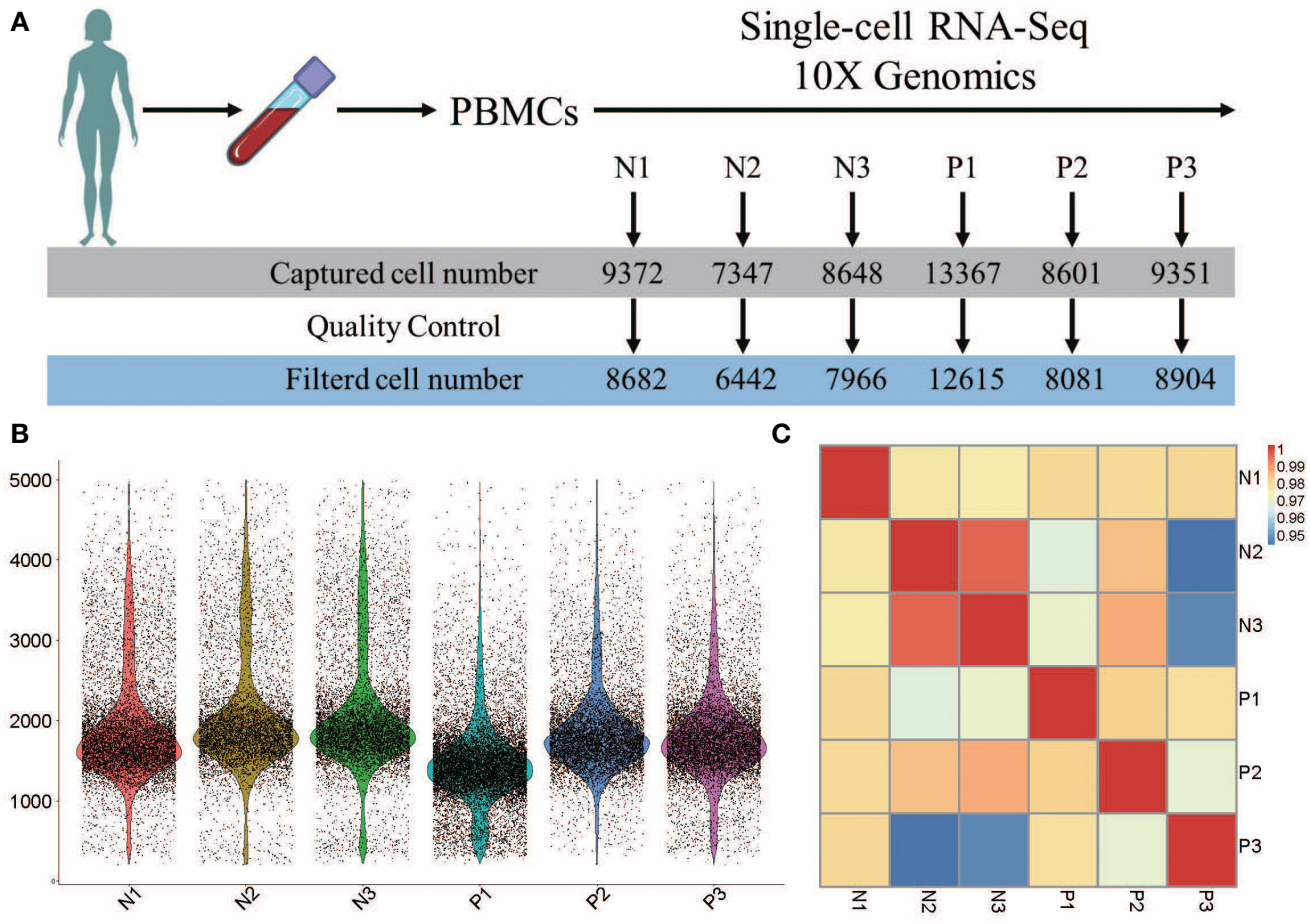
Heterogeneity of PBMC population analysi by single-cell RNA-seq. **(A)** The number of single cells in each sample caputured by single-cell RNA-seq 10X genomics. **(B)** The violin plot shows the gene number of each sample. **(C)** Pearson’s correlation plot visulizaing the correlation values between samples.

The number of captured cells in the 6 samples was 13367 for P1, 8601 for P2, 9351 for P3, 9372 for N1, 7347 for N2, and 8648 for N3, which respectively decreased to 12615, 8081, 8904, 8682, 6442, and 7966 after quality control (**Figure 1A**). Data from multiple sequencing were merged using a cell ranger pipeline, and all cells were sorted according to the number of detected genes. Approximately, 1710 genes were identified in these 6 samples, and more genes were identified in N3 (a normal control) than in other samples (**Figure 1B**).

Pairwise correlation values of inter-groups ranged from 0.94 to 0.99 (**Figure 1C**), revealing high consistency in cell type distribution among samples and inter-group heterogeneity.

### Dynamics of major PBMC immune cell types

**Figure 2:**
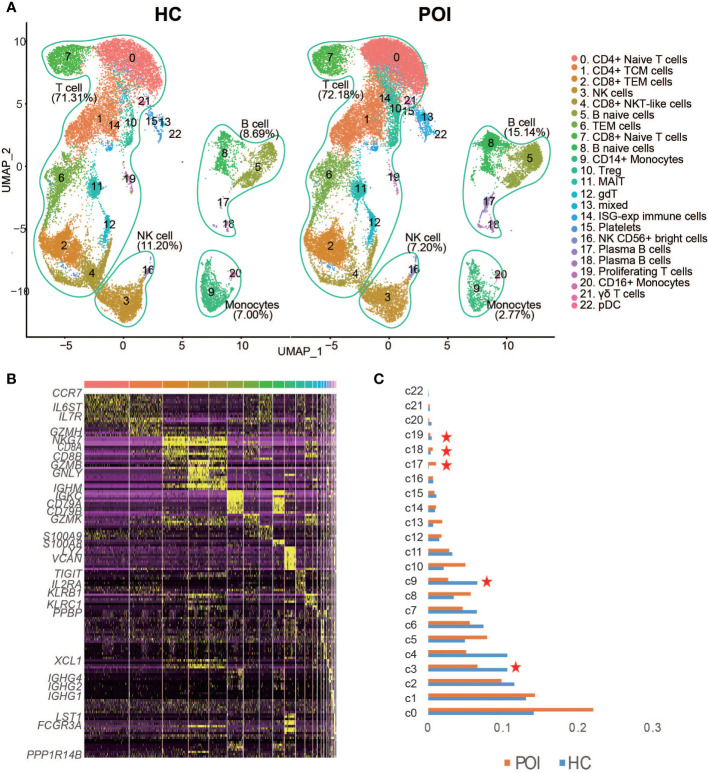
Single-cell transcriptional profiling of PBMCs from POI and HC. **(A)** Two- dimensional representation of cell types identified by scRNA-seq. **(B)** Heatmap of the top 10 marker genes from each cluster. **(C)** The proportion of each cluster in POI group compared with the HC group. Red star indicates statistical significance.

We then performed unsupervised clustering to separate the cells into 22 distinct clusters, which were visualized by Uniform Manifold Approximation and Projection for Dimension Reduction (UMAP) (**Figure 2A**). Based on the previously validated canonical marker genes, these clusters were assigned to 9 cell lineages, including T cell subsets (naive T cells, CD4+ T cells, naive CD8+ T cells, effector CD4+ T cells, and effector CD8+ T cells), B cell subsets (naive B cells and plasma B cells), NK cells, monocyte cells, and DCs. The distribution of these cells in HC and POI samples is shown in **Figure 2A**. T cells constituted the majority of all cells. B cells, NK cells, monocytes, and other cell types constituted small percentages of cells, It is consistent with the characteristics of the composition of human peripheral blood cells. The heatmap confirmed the distinct gene expression patterns among the clusters (**Figure 2B**).

As shown in **Figure 2C**, a significant decrease in the abundance of CD14+monocytes (p-value=0.0013, two-sided t-test) and proliferating T cells (p-value=0.047, two-sided t-test), and NK cells (p-value=0.035, two-sided t-test) was identified in POI compared with HC. In contrast, the abundance of plasma B cells showed a significant increase (p-value=0.0057, two-sided t-test) in POI. No significant difference was observed in the abundance of CD4+ T cells (p-value=0.066, two-sided t-test), while a significant reduction was observed in the abundance of CD8+ T cells in patients with POI (p-value=0.02, two-sided t-test). The CD4/CD8 ratio was significantly higher in patients with POI. Moreover, three atypical cell types, including CD8+ NKT-like cells, MAIT cells, and γδ T cells, were decreased in POI patients; however, results were not statistically significant. Notably, the abundance of Treg cells was increased in patients with POI.

### DEGs from POI predominantly expressed in B and monocyte cells

**Figure 3:**
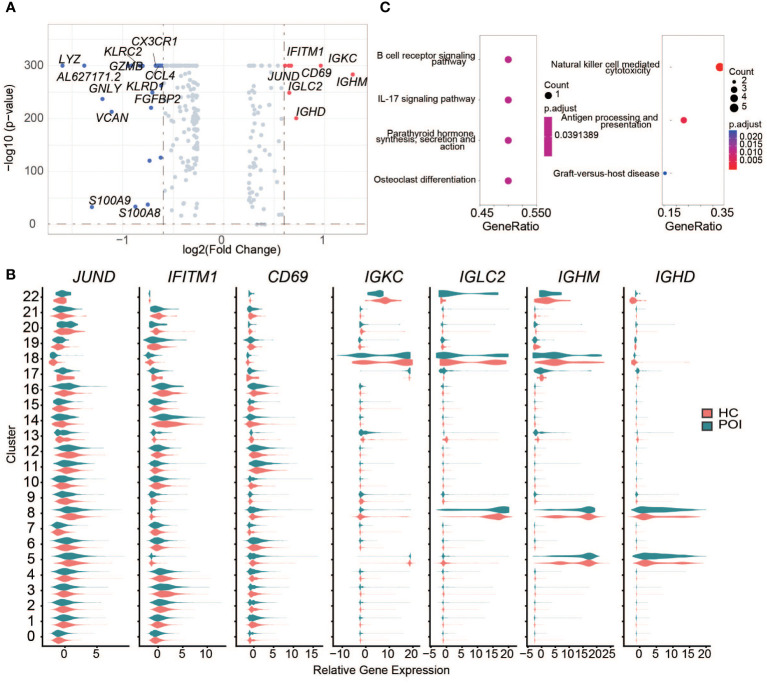
DEGs between POI and HC across all the clusters. **(A)** The DEGs between POI and HC samples. The significantly down-regulated and up-regulated genes were colored with blue and red, respectively. **(B)** The expression levels of up-regulated genes in each cluster. **(C)** Dot plot of the enriched KEGG pathways for up-regulated (left) and down-regulated (right) genes.

In total, 24 downregulated genes and 7 upregulated genes between HC and POI were found in PBMCs (**Figure 3A**). *IGHM* and *LYZ* were respectively the most significantly upregulated and downregulated genes among all cell clusters of POI patients. Among the upregulated genes, *IGHM, IGKC, IGHD*, and *IGLC2* were predominantly expressed in B cells and DCs of POI patients corresponding to clusters 5, 8, 17, 18, and 22 (**Figure 3B**). *IGKC, IFITM1, CD69*, and *JUND* were upregulated in almost all cell types of POI patients compared with HC (**Figure 3B**). In contrast, downregulated genes, such as *LYZ, GNLY*, *VCAN*, and *S100A9*, were mostly related to NK cells, CD8+ T cells, and monocytes (**Figure S1**).

To further investigate the transcriptomic differences between HC and POI, pathway enrichment analysis was performed using the DEGs. Upregulated genes were enriched in B cell receptor signaling pathway, IL-17 signaling pathway, parathyroid hormone synthesis, secretion and action, and osteoclast differentiation pathways. Downregulated genes were mainly enriched in NK cell-mediated cytotoxicity, cytolysis, and cell-killing functions (**Figure 3C**).

### Monocyte cell exhaustion in POI

**Figure 4:**
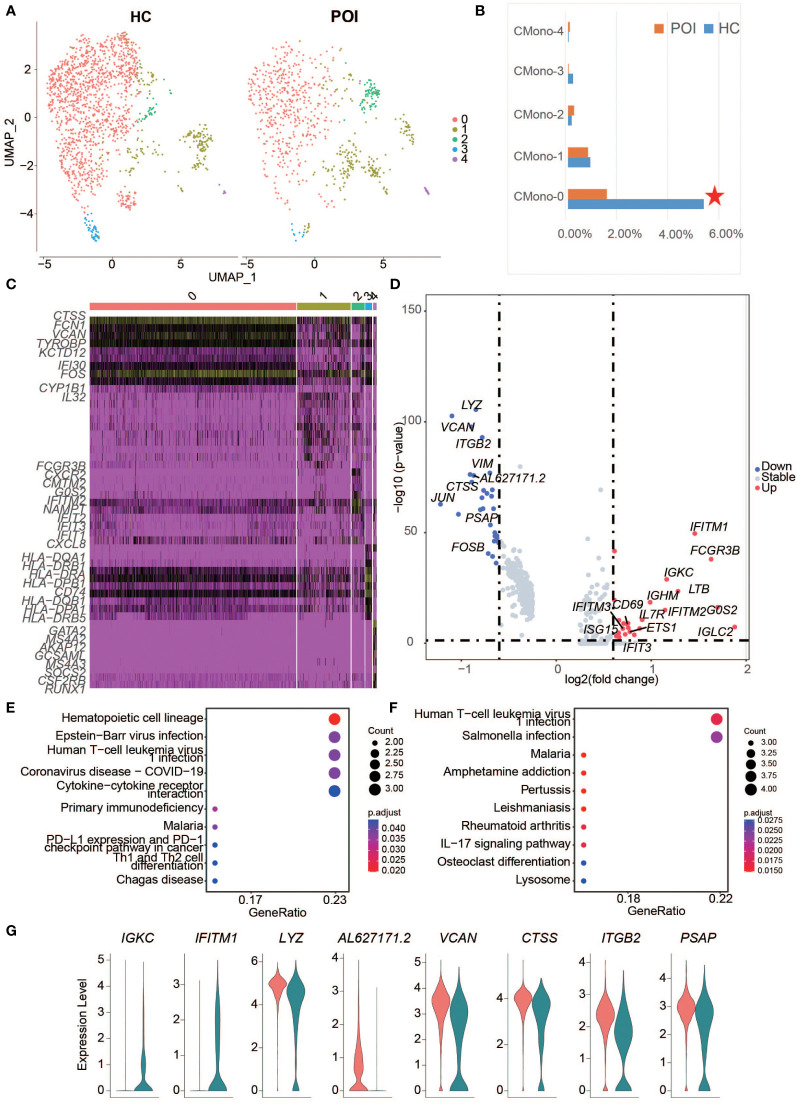
Single-cell expression analysis of monocytes in POI and HC. **(A)** UMAP plot showing five cell subclusters from the monocyte cells in POI and HC. **(B)** The proportion of each subclusters of monocytes in POI and HC, Red star indicates statistical significance. **(C)** The heatmap of top 10 marker genes in each subclusters of monocytes. **(D)** The DEGs of monocytes between POI and HC. **(E, F)**. Dot plot of enriched KEGG terms of the down-regulated **(E)** and up-regulated genes **(F, G)** Violin plots for some marker genes in classical monocytes.

The results of DEG analysis for all clusters showed a decrease in monocytes, especially CD14+ monocytes, suggesting that monocytes may be affected in POI. Therefore, we re-analyzed the cluster of monocytes and divided cells into 5 subsets (**Figure 4A**), including CMono0, CMono1, CMono2, CMono3, and CMono4 (**Figure 4B**). Surprisingly, we found that the proportion of Mono0-subset showed a significant decrease while the proportion of other subsets remained stable.

Each subset exhibited a distinct gene expression pattern. CMono0, expressed high levels of classical monocyte marker genes, including *CTSS, FCN1, VCAN, KTD12*, *CYP1B1*, and *IFI30*, indicating that this subset was predominantly classical monocytes. CMono2 expressed high levels of neutrophil-related genes, including *CXCL8, IFIT3, IFIT2, IFIT1, NAMPT, IFITM2, G0S2, FCGR3B, CXCR2*, and *CMTM2.* CMono3 expressed high levels of DC marker genes, while CMono4 expressed high levels of basophil marker genes (**Figure 4C**). All cells were phagocytes and there was a group of innate myeloid immune cells.

Next, 47 DEGs from all subsets of monocyte were identified between HC and POI. The highly up-regulated DEGs, such as IGLC2, G0S2, FCGR3B, IFITM1, LTB, IGKC, and IFITM2, were predominantly found in classical monocytes in POI (**Figure 4D**). These DEGs were significantly enriched in the infection and T cell differentiation pathways (**Figures 4E, F**). Interestingly, *ISG15*, a gene from these pathways, was highly upregulated in the CMono1-subset.

Interestingly, 8 DEGs from the classical monocytes, including 2 upregulated genes (*IGKC* and *IFITM1*) and 6 downregulated genes (*LYZ, AL627171.2, VCAN, CTSS, ITGB2*, and *PSAP*) also appeared in the DEGs from all cell clusters, indicating that classical monocytes were the main driving component for these DEGs (**Figure 4G**). Our results were consistent with those from previous studies. For example, a recent study suggested that the classical monocytes overexpress interferon-induced transmembrane protein 1 in perinatal hepatic inflammation (29), which is a highly specific immune marker of normal endometrial stroma (30).

### NK cells in POI

NK cells are an important group of immune cells, which are widely distributed in human peripheral lymphoid organs and in the circulatory system.

**Figure 5:**
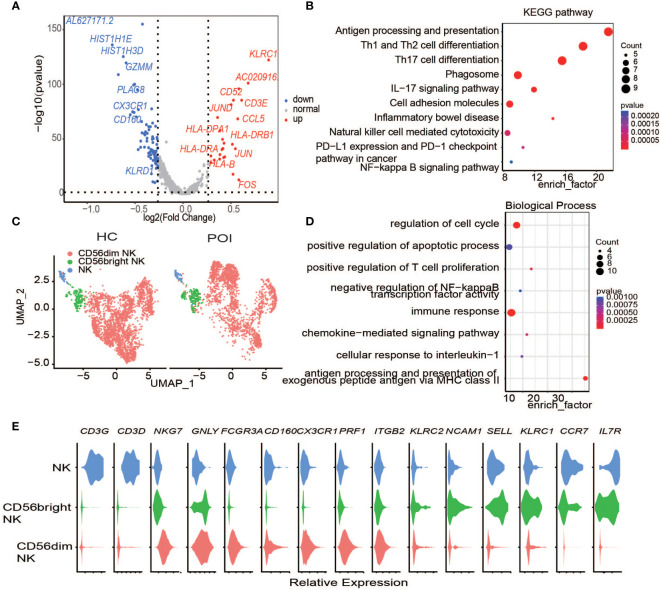
Single-cell expression analysis of Natural Killer cells in POI and HC. **(A)** The DEGs of NK cells between POI and HC. **(B)** Dot plot of enriched KEGG terms of the DEGs. **(C)** UAMP plot showing the NK cell subclusters in POI and HC. **(D)** Dot plot of enriched Biological of the DEGs. **(E)** Violin plots for classical markers of NK cell clusters.

CD56Dim,CD56bright is the main subtype of NK cells in the blood, and CD16+CD56Dim is the main subtype of NK cells in peripheral blood. In our dataset, two main groups of NK cells have been identified, namely c3 and c16 (**Figure 2A**). They are also named CD16^bright^ CD56^Dim^ and CD16^Dim^ CD56^bright^ according to their markers (**Figures 5C, E**). The average proportions of the two clusters in HC were 10.5% and 0.65%, while in POI, their proportions decreased to 6.6% and 0.64%.

Next, we investigated the DEGs in NK cells between POI and HC. In total, 19 upregulated genes and 8 downregulated genes were identified (**Figure 5A**). KLRC1, which encodes the killer cell lectin-like receptor C1 and is preferentially expressed in NK cells, was the most upregulated DEG in the NK cells of POI patients. This protein forms a complex with another family member, *KLRD1/CD94*, and has been implicated in the recognition of the MHC class I HLA-E molecules in NK cells. *CD3E* was another highly upregulated gene. It is a cell surface protein involved in signal transduction to T cell antigen receptor complex; therefore, it plays a crucial role in the adaptive immune response. Enrichment analysis showed that the differentially expressed genes were enriched primarily in the IL-17 signaling pathway, cellular adhesion molecules, and in pathways related to NK cell-mediated cytotoxicity. (**Figure 5B**). Differential gene expression is responsible for the regulation of biological functions such as cell cycle progression, apoptosis, and immune response (**Figure 5D**).

In addition, the upregulation of chemokine CCL5, together with the upregulation of HLA-DPA1, HLA-DRB1, and HLA-DRA suggested that compared with HCs, NK cell-mediated response and cytotoxicity were notable in patients with POI (**Figure 5A**) (31).

### Plasma B cells in POI

B cells are the second-largest type of lymphocytes in humans and mainly participate in humoral immunity. In human peripheral blood, B cells differentiate mainly into the memory B cells and plasma cells. Plasma cells, also known as effector B cells, mainly secrete antibodies and cytokines.

**Figure 6:**
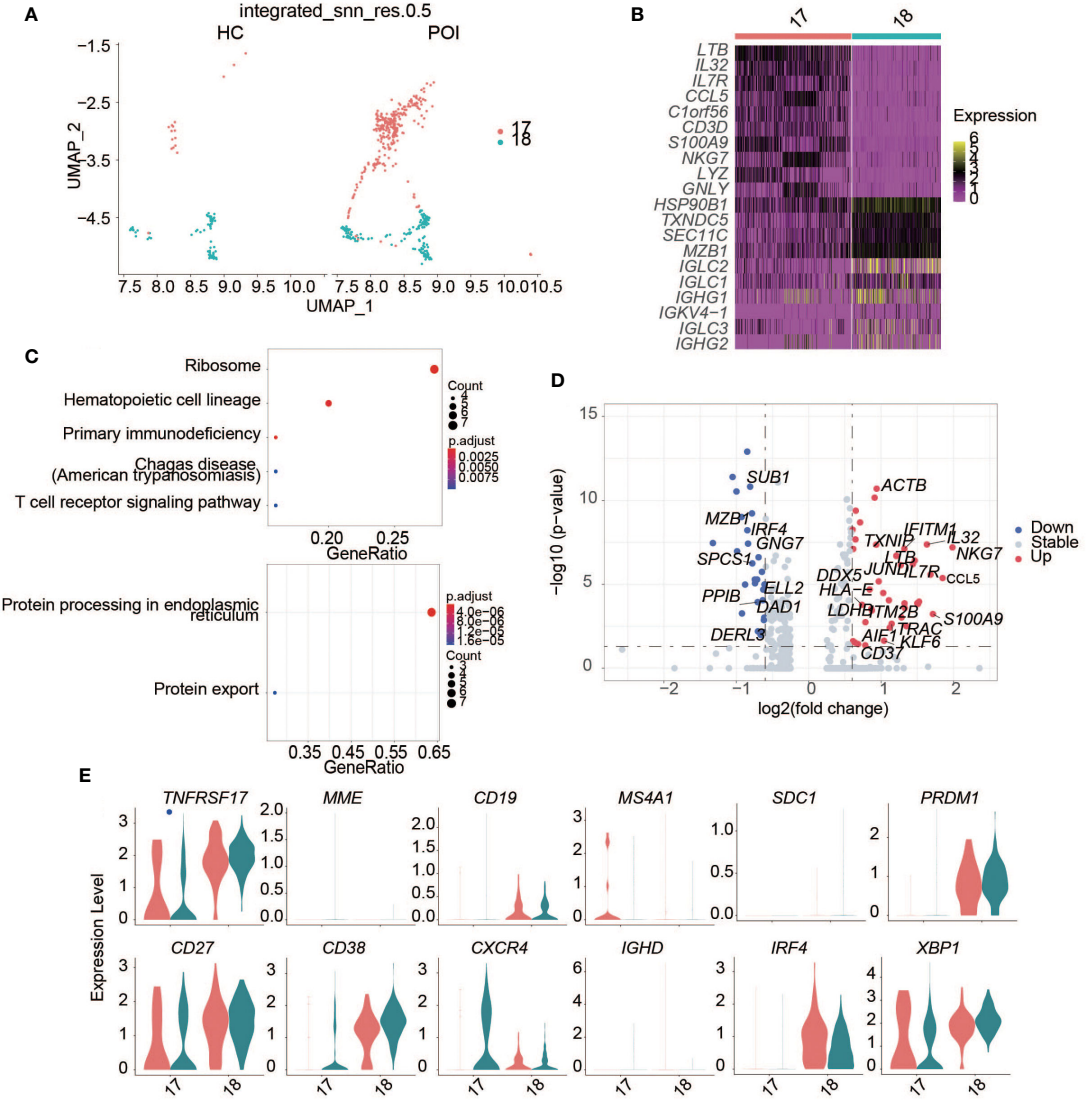
Single-cell expression analysis of plasma B cells in POI and HC. **(A)** UAMP plot showing the plasma B cell clusters in POI and HC. **(B)** The heatmap of top 10 marker genes in each plasma cell cluster. **(C)** Dot plot of enriched KEGG terms of the up-regulated (above) and down-regulated genes (below). **(D)** The DEGs of plasma B cells between POI and HC. **(E)** Violin plots of plasma B cell markers.

A significantly higher abundance of plasma cells was observed in POI compared with HC (**Figures 6A, B**). Plasma cells were divided into two clusters, namely c17 and c18. In humans, B cells can be divided into naive mature B cells (CD19+CD27-) and memory B cells (CD19+CD27+) based on CD19 and CD27 expression. After antigen stimulation, mature B cells differentiate into either memory B cells or plasma cells. Mature B cells lose some of their surface antigens upon differentiation into plasma cells. We found that B cell markers, including CD19 and MS4A1 (an encoding CD20), were markedly downregulated, while the plasma cell marker, CD38, was highly expressed (**Figure 6E**).

We identified DEGs related to plasma cells between patients with POI and HCs (**Figure 6D**). In patients with POI, S100A9, and CCL5 were upregulated significantly (**Figure 6D**). Enrichment analysis showed that the upregulated genes were mainly enriched in the ribosome, primary immunodeficiency, and T cell receptor signaling pathways, while downregulated genes were mainly enriched in protein processing in the endoplasmic reticulum and protein export (**Figure 6C**).

### Treg cells in POI

Treg cells play a key role in maintaining self-tolerance and preventing autoimmune diseases. Previous data showed that Treg abundance is decreased in patients with POI (32).

**Figure 7:**
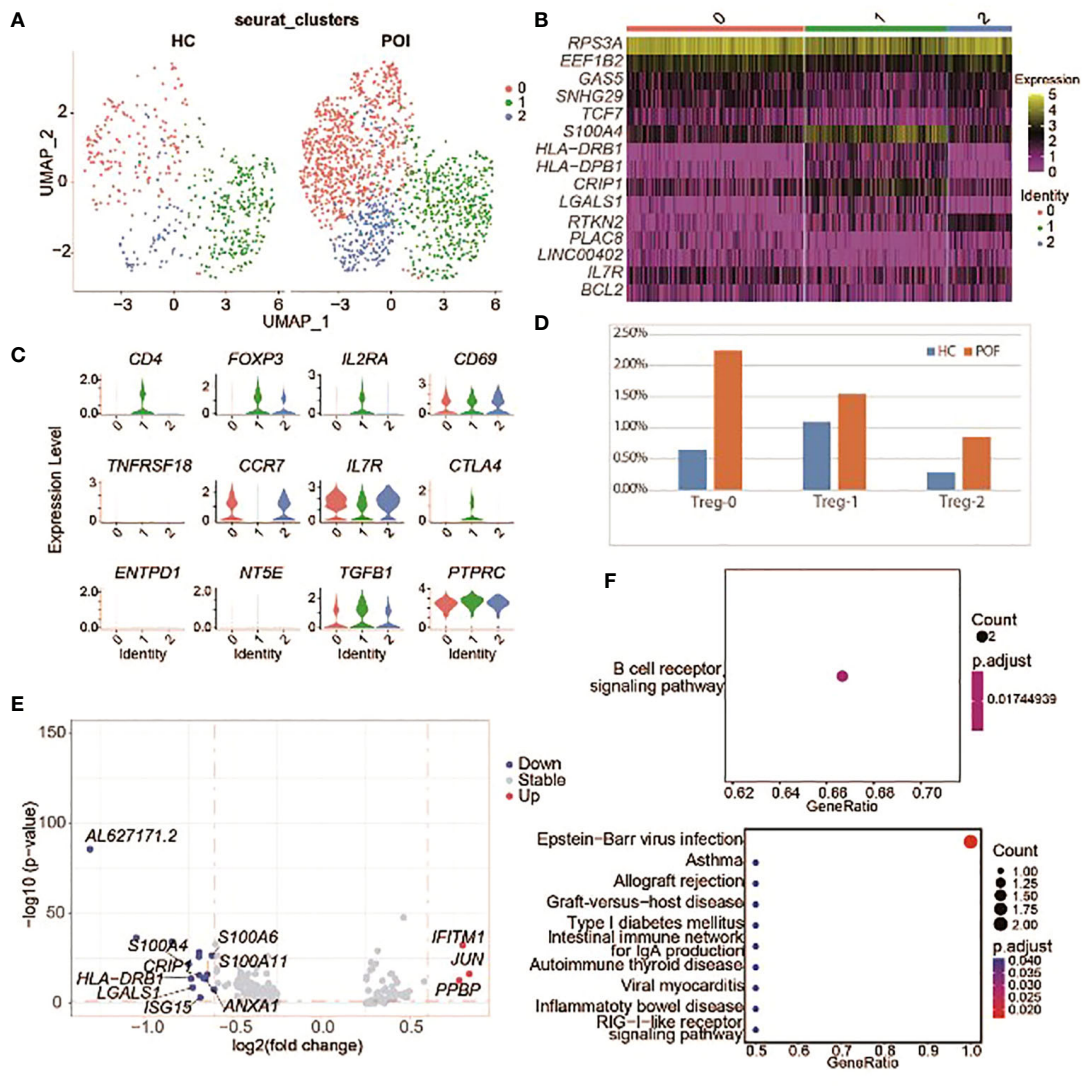
Single-cell expression analysis of Treg cells in POI and HC. **(A)** UAMP plot showing the Treg cell subclusters in POI and HC. **(B)** The heatmap of top 5 marker genes in each Treg cell subclusters. **(C)** Violin plots for marker genes of Treg cell subcluster. **(D)** The proportion of Treg cell subclusters between POI and HC. **(E)** DEGs of Treg cell subclusters between POI and HC. **(F)** Dot plot of enriched KEGG terms of the up-regulated (above) and down-regulated (below) genes.

In this study POI samples were labeled with CD4- CD25+ FOXP3+ and a stable expression of FOXP3 gene. Then, we reanalyzed Treg cells and divided them into 3 subsets (**Figures 7A, B**), including Treg-0, Treg-1, and CD4^-^ FOXP3^+^ Treg cells (**Figure 7C**). All Treg cell subsets displayed a higher proportion in POI (**Figure 7D**).

DEGs from Treg cells between patients with POI and HCs were identified (**Figure 7E**). Only three genes were upregulated in POI, including *IFITM1, JUN*, and *PPBP*. Enrichment analysis showed that the upregulated genes were mainly enriched in B cell receptor signaling pathway, while the downregulated genes were mainly enriched in immune pathways including allograft rejection, autoimmune thyroid disease, and RIG−I−like receptor signaling pathway (**Figure 7F**).

### Dynamics of the intercellular communication networks in POI

**Figure 8:**
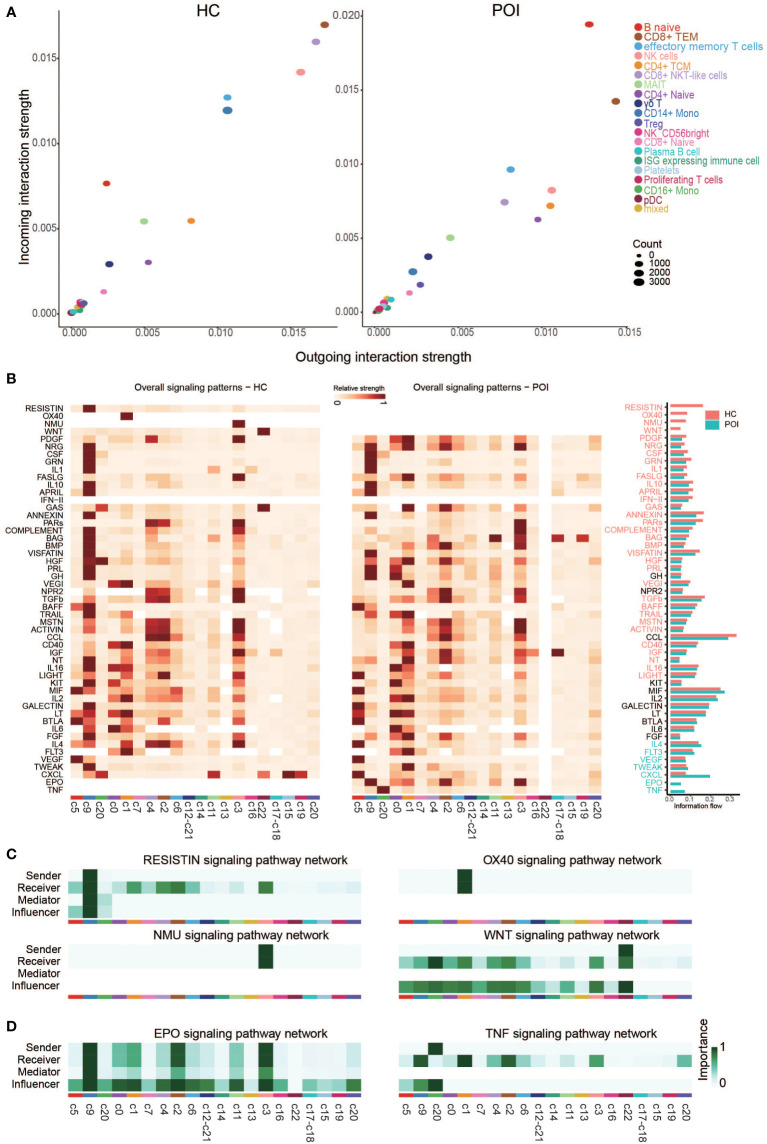
Cross talk between the cell clusters in POI and HC. **(A)** Projecting cell clusters onto a two-dimensional manifold according to their functional similarity in POI and HC. Each dot represents the communication network of one cluster. Dot size is proportional to the overall communication probability. **(B)** Heatmap of the signals contributing most to outgoing or incoming signaling of certain cell groups in POI and HC. **(C, D)** Heatmap of the inferred intercellular communication network of the unique signaling pathways in HC **(C)** and POI **(D)**.

We compared the outgoing and incoming interactions strength to identify cell populations with significant alterations in sending or receiving signals between HC and POI. We observed that naive B cells and classical monocytes were the major affected sources and targets in patients with POI compared with HC (**Figure 8A**). Naive B cells had higher levels of outgoing and incoming interaction strength, whereas classical monocytes showed reduced levels of cell communication, suggesting an enhanced innate immune response and functional impairment of classical monocyte in POI. Besides, CD8+ NKT-like cells and NK cells showed markedly reduced levels of outgoing and incoming interaction strength. These findings are consistent with autoimmune ovarian damage caused by an increased abundance of autoantibody-producing B cells (33). Similarly, our results are consistent with defective monocyte polarization and impaired NK cell activity in POI reported by Hoek et al. (23). We subsequently compared the overall relative strength of the identified signaling pathways in each cell cluster by its aggregated outgoing and incoming signals (**Figure 8B**). Significant signaling pathways were ranked based on differences in the overall communication probability in the indicated networks between HC and POI. Four pathways (RESISTIN, OX40, NMU, and WNT) were unique in HC, while 2 pathways (EPO and TNF) were unique in POI (**Figures 8C, D**). In addition, there were 29 pathways significantly enriched in HC, and 5 pathways significantly enriched in POI (**Figure S2**). Among them, some pathways that are normally originating from classical monocytes, such as COMPLEMENT and BMP, were originating from NK cells in POI. In contrast, the receivers of some pathways, such as APRIL, LIGHT, and IL4, changed from monocytes to NK cells and naive B cells. Taken together, these results strongly indicate that the intercellular communication networks among the cell clusters were largely disrupted in POI.

## Discussion

POI is a heterogeneous and multifactorial syndrome in which idiopathic cases account for 74% to 90% of all cases of POI, and the etiology is still unknown (34). Current studies suggest that the immune system plays a crucial role in POI and that the human ovary is a common target for autoimmune attack. Although accumulating evidence indicates that POI is associated with alterations in cellular and humoral immunity (35), the precise mechanism underlying idiopathic POI remained unclear.

Previous studies have demonstrated that there are three main types of POI based on its pathogenesis, including inadequate oogenesis, oocyte depletion/atresia, and follicle dysfunction (36). Although many candidate genes of POI have been identified based on NGS, few have been functionally verified to be associated with the pathogenesis of POI (19). However, the immune-related genes that induce POI have not been reported. Notably, we identified a series of DEGs, such as *IGHM, IGKC, GNLY, LYZ, S100A9*, and *CCL5*. The *IGHM* gene encodes the C region of the mu heavy chain, which defines the IgM isotype. Naïve B cells express the transmembrane forms of IgM on their surface. Using somatic recombination or isotype switching, activated B cells can switch to express individual downstream heavy chain C region genes. It was reported that IgM is predominant in autoimmune diseases (37). Based on our data, *IGHM* was upregulated in naive B cells, circulating plasma cells, and pDC cells. *IGKC* has been validated as a prognostic and therapeutic biomarker in human breast cancer and other cancers (38). *LYZ* gene encodes human lysozyme, which is a crucial biodefense effector in innate immunity and has lytic activity against bacterial peptidoglycan, thereby protecting the host from pathogenic infection (39). *GNLY* encodes an antimicrobial protein, which exists in the granules of human cytotoxic T lymphocytes and NK cells. Consistent, our result showed that GNLY is mainly expressed in T cell and NK cell clusters in HC and POI patients. *GNLY* is the first identified lymphocyte-derived alarmin capable of promoting APC recruitment, activation, and antigen-specific immune response (40). Altogether, these results suggest that the DEGs in POI, including *IGHM, IGKC, GNLY*, and *LYZ*, may be potential markers and targets for the diagnosis and prevention of POI.

Previous study indicated that S100A9 can activate NLRP3-induced inflammatory corpuscle formation and lead to cell pyroptosis by regulating TLR4. Recent studies found that TLR4 is highly expressed in primary follicles and granulosa cells independent of luteinizing hormone (41). These findings suggest that follicular granulosa cells may be the target of S100A9, and S100A9 can induce the pyroptosis of granulosa cells and premature ovarian failure through TLR4. In addition *CCL5* can bind to its cell surface receptor chemokine receptor type 5 (CCR5). The transcriptome analysis of bovine follicles revealed that CCR5 is expressed in granulosa cells and theca cells (42). Previous studies suggested that the progression of ovarian dysfunction is associated with immune dysregulation induced by an abnormal proportion of CD8+ T cells and upregulation of CCL5 and IFN-γ (43). Circulatory CCL5 can bind to CCR5 on the surface of granulosa cells, further promoting the pyroptosis of granulosa cells, reducing the number of follicles, and ultimately leading to POI.

Compared with HCs, the percentage of monocytes was significantly decreased in POI patients.It has been shown that the dysfunction of monocytes and dendritic cells is part of deregulated cell-mediated immunity (44). A previous study described the decreased expression of adhesion molecules in a subpopulation of monocytes from the peripheral blood of patients with diabetes mellitus (45). Follicular growth initiation is characterized by cuboidalization of flattened granulosa cells. Adhesive junctions such as N-cadherin and nectin can regulate this process (46). Gap junctions provide intercellular communication among different types of cells in the ovary, which is critical for proper ovarian function (47). The expression of adhesion molecules is the main characteristic of large monocytes (48). In addition, NK cells are a crucial component in maintaining immunity. despite their importance, the role of NK cells remains poorly understood in the reproductive processes, such as implantation, trophoblast invasion, and spiral artery remodeling. The relationship between the number of NK cells and infertility remains controversial. Although a decreased number of NK cells was observed in POI patients, the genes associated with NK cell activity were highly expressed in our study. Some researchers support the association between the number of NK cells in the peripheral blood and infertility, while others claim that NK cells activation needs also to be examined (49). B lymphocytes produce antibodies and play an important role in humoral immunity (50). In our study, the number and cytotoxicity of NK cells were significantly reduced and the number of plasma cells was significantly increased in POI. Furthermore, the genes that were significantly upregulated in POI were predominantly found in B cells. In addition, DEGs in NK and B cells were both enriched in IL-17 signaling pathway, which plays crucial roles in aggravating inflammation. The inflammatory response affects steroid-producing cells in the pre-ovulatory follicles, particularly internal and external theca layers, corpora luteum, and granulosa cells (42). It suggests that NK cells and B cells in POI may regulate the inflammatory response by changing the number of cells or affecting the expression of cytokines such as IL-17. Various treatment strategies have been applied due to the complexity of POI, and stem cell transplantation has been the most effective method. Modulation of monocytes, NK cells, and B cells may also serve as a simple and efficacious immunotherapy strategy for POI.

Previous studies indicated that the number of CD4+CD25+FOXP3+ Treg cells is reduced in the peripheral blood of patients with POI (15). However, this is not consistent with our result, and several reasons contribute to this inconsistency. First, the age of patients with POI in our study was higher than that in other studies. Previously, it has been demonstrated that the number of Treg cells in lymphoid organs increases with age (51). Furthermore, naive B cells can convert CD4+CD25- cells into CD4+CD25+Foxp3- Treg cells (Treg-of-B cells) (52). Second, Xiong et al. reported that 20% of POI patients were positive for antithyroid peroxidase antibody (anti-TPO) and/or anti-thyroglobulin antibody (anti-TG), while in our study, POI patients were negative for anti-TPO and/or anti-TG. Therefore, Treg lymphopenia may be not a reliable marker for the development of premature ovarian failure.

By comparing different signaling pathways in each cell cluster between HCs and patients with POI, TNF pathway was unique in POI and classical monocytes were the main receivers and senders of TNF signaling pathway in POI. TNF-α mainly originates from monocytes, macrophages, DC cells, activated B lymphocytes, and T lymphocytes. It can bind to two receptors (TNFR1 and TNFR2) and has pleiotropic effects on immunity and inflammation (53). TNF-α plays a critical role in several signaling pathways, including NF-κB, JAK/STAT, Akt, p38/MAPK, ERK, and Wnt/β-catenin signaling pathways (34). Many studies on POI have measured TNF-α and IL-1β levels as indexes of inflammation. IFN‐γ and TNF‐α directly lead to granulosa cell dysfunction and contribute to follicle atresia and ovarian insufficiency (13).

In summary, this is the first study that uses single-cell sequencing to characterize different subsets of peripheral blood lymphocytes in patients with POI. Compared with the HC group, the proportion of classical monocytes and NK cells was decreased, and the proportion of plasma B cells was increased. DEGs in NK and B cells were both enriched in IL-17 signaling pathway, which plays crucial roles in inflammation. Furthermore, the TNF pathway was found to be unique in POI, with classical monocytes being the major receivers and senders of TNF signaling. Taken together, these findings suggest that abnormalities of peripheral blood immune cells may be involved in the etiology of idiopathic POI, and POI may benefit from targeting monocytes, NK, and B cells. However, the limited sample size of this study prevents the generalizability of results. Therefore, experiments and clinical trials with larger sample sizes are needed.

## Data availability statement

The datasets presented in this study are deposited in the GeneBank repository, accession number PRJNA923721.

## Ethics statement

The studies involving human participants were reviewed and approved by the Institutional Review Board of Changhai Hospital. The patients/participants provided their written informed consent to participate in this study.

## Author contributions

CZ, DY and HG provided the initial concept and experimental design. JH and QL conducted the literature review, SH and JH collected patient samples, SQ and YM performed single cell RNA sequencing. DY performed the data analysis of single cell RNA sequencing. CZ, DY and YM drafted the manuscript. HG, SL and JH revised the manuscript. All authors provided critical revisions. All authors contributed to the article and approved the submitted version.

## Supplementary material

Supplementary Figure 1 Violin plots of significantly down-regulated genes in POI and HC.

Supplementary Figure 2 Heatmap of the inferred intercellular communication networks of 5 signaling pathways with significant differences between HC (left) and POI (right).
