# Supplementary figures and images for: Single-cell RNA sequencing of peripheral blood reveals immune cell dysfunction in premature ovarian insufficiency
Source: Front Endocrinol (Lausanne). 2023 May 8;14:1129657. doi: 10.3389/fendo.2023.1129657 (PMC10200870; doi:10.3389/fendo.2023.1129657)

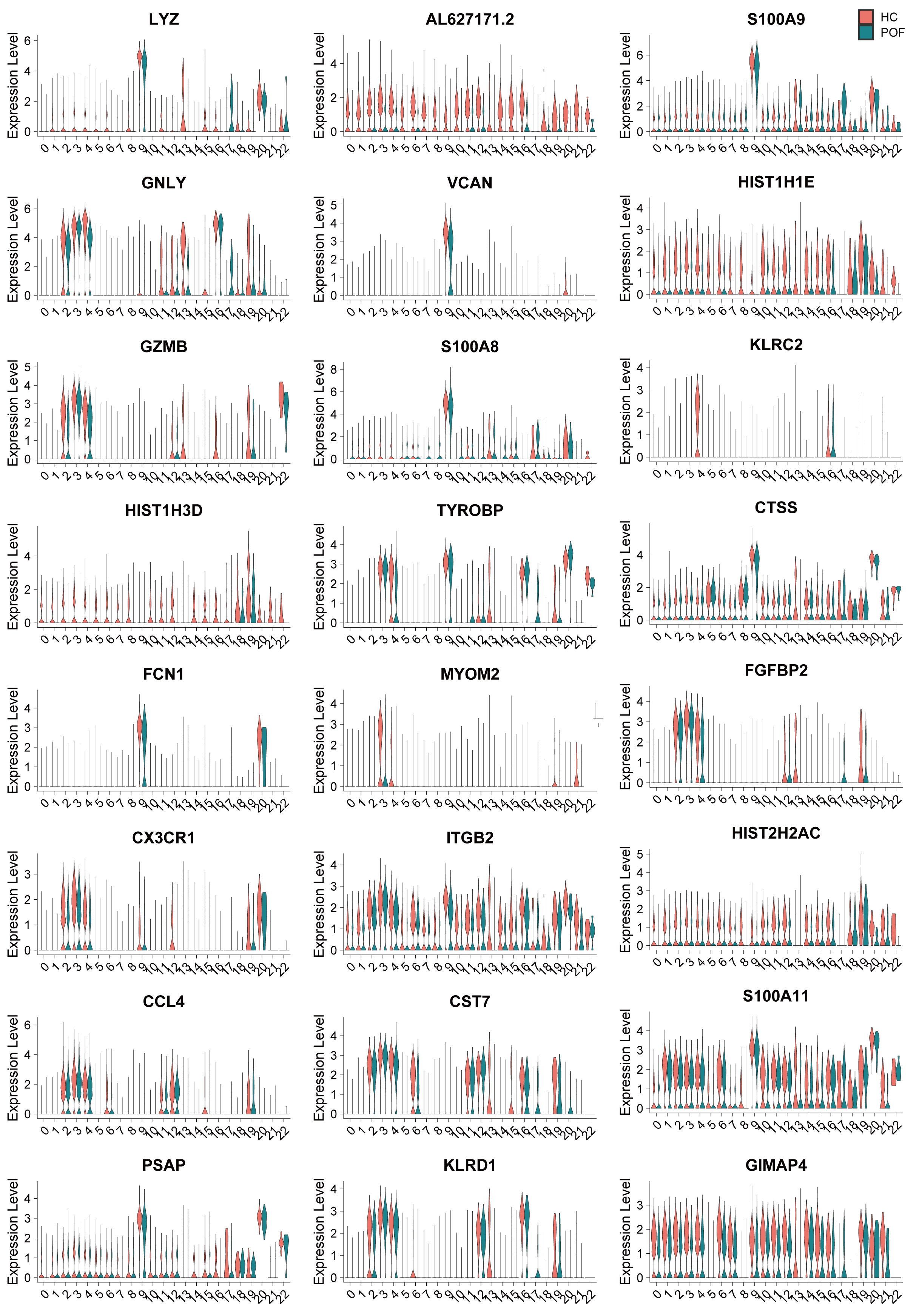

Supplement: Supplementary Figure 1 — Violin plots of significantly down-regulated genes in POI and HC. [file Image_1.jpeg]

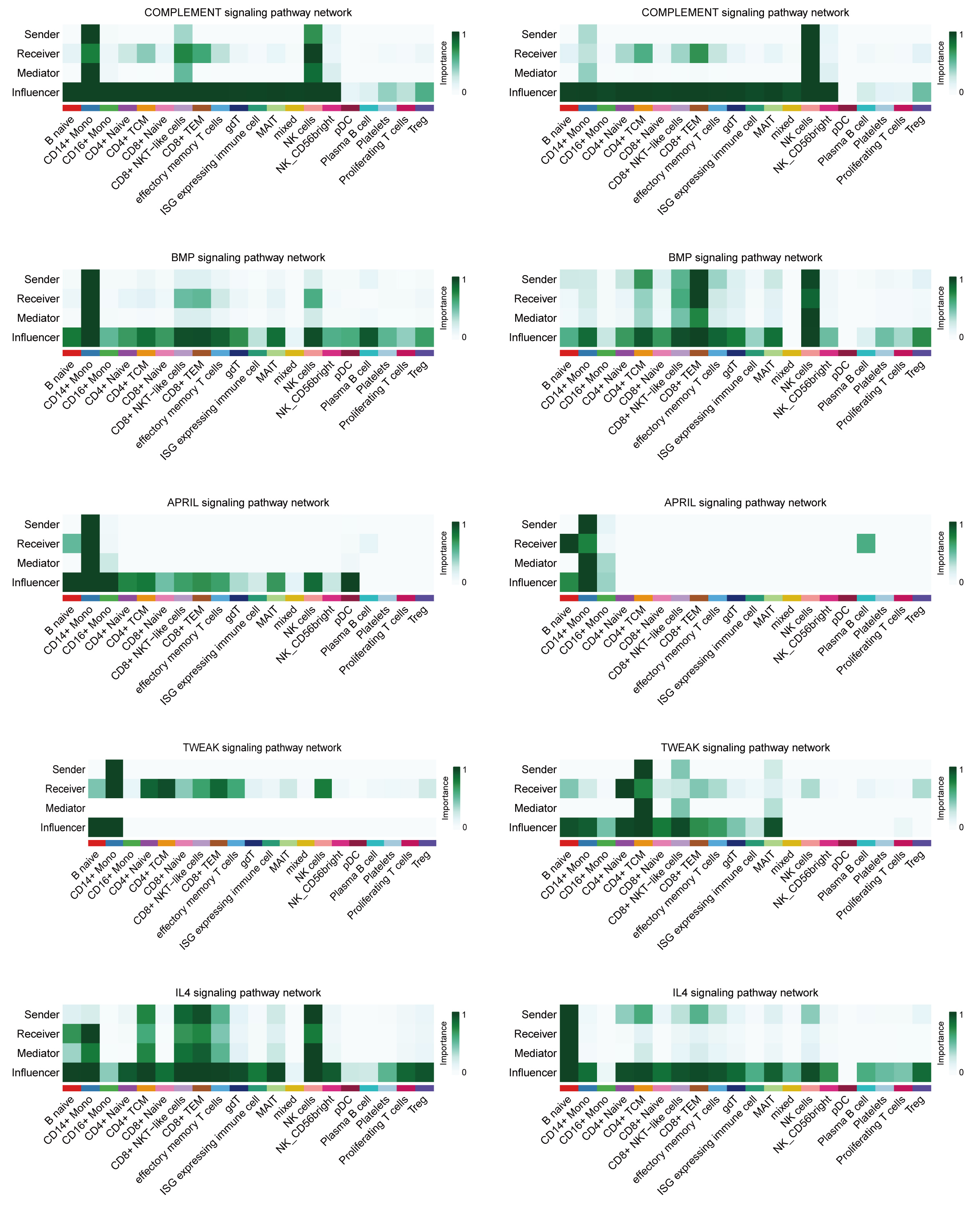

Supplement: Supplementary Figure 2 — Heatmap of the inferred intercellular communication networks of 5 signaling pathways with significant differences between HC (left) and POI (right). [file Image_2.jpeg]
